# Empirical comparison of cross-platform normalization methods for gene expression data

**DOI:** 10.1186/1471-2105-12-467

**Published:** 2011-12-07

**Authors:** Jason Rudy, Faramarz Valafar

**Affiliations:** 1 Biomedical Informatics Research Center, San Diego State University, 5500 Campanile Dr, San Diego, CA, USA

## Abstract

**Background:**

Simultaneous measurement of gene expression on a genomic scale can be accomplished using microarray technology or by sequencing based methods. Researchers who perform high throughput gene expression assays often deposit their data in public databases, but heterogeneity of measurement platforms leads to challenges for the combination and comparison of data sets. Researchers wishing to perform cross platform normalization face two major obstacles. First, a choice must be made about which method or methods to employ. Nine are currently available, and no rigorous comparison exists. Second, software for the selected method must be obtained and incorporated into a data analysis workflow.

**Results:**

Using two publicly available cross-platform testing data sets, cross-platform normalization methods are compared based on inter-platform concordance and on the consistency of gene lists obtained with transformed data. Scatter and ROC-like plots are produced and new statistics based on those plots are introduced to measure the effectiveness of each method. Bootstrapping is employed to obtain distributions for those statistics. The consistency of platform effects across studies is explored theoretically and with respect to the testing data sets.

**Conclusions:**

Our comparisons indicate that four methods, DWD, EB, GQ, and XPN, are generally effective, while the remaining methods do not adequately correct for platform effects. Of the four successful methods, XPN generally shows the highest inter-platform concordance when treatment groups are equally sized, while DWD is most robust to differently sized treatment groups and consistently shows the smallest loss in gene detection. We provide an R package, CONOR, capable of performing the nine cross-platform normalization methods considered. The package can be downloaded at http://alborz.sdsu.edu/conor and is available from CRAN.

## Background

Simultaneous measurement of gene expression on a genomic scale can be accomplished using microarray technology or by sequencing based methods [1-3]. Many high-throughput mRNA expression experiments produce data that can be of value to other researchers when analyzed in new contexts or in combination with data from other experiments. In particular, the statistical power and reproducibility of gene expression studies can be increased by combining data across multiple studies [4-6]. While next generation sequencing seems likely to replace microarrays for expression analysis in the near future, the large amount of microarray data already in existence could continue to be useful to researchers for many years to come.

Modern microarrays are commercially produced, and one-color hybridization schemes are often employed. Several companies have emerged as leading manufacturers, each using different manufacturing techniques, labeling methods, hybridization protocols, probe lengths, and probe sequences. Table 1 lists some important characteristics of the microarray platforms analyzed in this work. These characteristics can affect microarray performance [7-10]. The length of probes represents a tradeoff between sensitivity and specificity, with longer probes being generally more sensitive and shorter being more specific. The use of linkers to reduce steric hindrance, as employed by the Applied Biosystems and Illumina platforms in table 1 is one method for increasing the sensitivity of short probes. The method by which probes are constructed and attached, and the overall construction of the array, can affect probe uniformity and intra-platform reproducibility. Labeling and detection chemistry affect the dynamic range of detection.

**Table 1:** Characteristics of relevant microarray platforms

Manufacture	Platform	Probe Length	Probe Type	Probe Construction	Number of Probes	Label Type	Detection method
Applied Biosystems	Human Genome Survey Microarray v2.0	60	DNA oligonucleotide with 3' carbon spacer to reduce steric effects	Presynthesized and contact spotted	32878	Digoxigenin (DIG) UTP	anti-DIG phosphatase catalyzed Chemiluminescence
Affymetrix	HG-U133 Plus 2.0 GeneChip^®^	25	DNA oligonucleotide	*In situ *photolithography	54675	Biotin	phycoerythrin-streptavidin-antibody fluorescence
Agilent	Whole Human Genome Oligo Microarray, G4112A	60	DNA oligonucleotide	*In situ *inkjet printing	43931	Cy3 or Cy5	Cy3 or Cy5 fluorescence
Illumina	Human-6 BeadChip, 48K v1.0	50	DNA oligonucleotide with 29 base address sequence as linker	Presynthesized, immobilized on beads, and randomly deposited in wells	47293	Biotin	strepatavidin-Cy3 fluorescence

Characteristics of microarray platforms analyzed in this work. See references [7-10] for information sources.

Chemiluminescence provides greater sensitivity for low levels of expression compared to fluorescence, but at the risk of saturation for highly expressed genes. The Applied Biosystems scanning procedure attempts to mitigate scanner saturation by using both a short and a long exposure to extend the dynamic range of its expression measurements. Probe sequences affect the binding constants between probes and target and non-target molecules, and therefore the sensitivity and specificity of each probe depends partially on its sequence. Salinity and composition of the hybridization solution, temperature, and incubation time of hybridization may also affect sensitivity and specificity. Data from two microarrays are directly comparable only if those microarrays are identical in all design parameters including probe sequences and have been subjected to similar hybridization conditions. Because no two platforms share the same set of probe sequences, no two platforms produce data that are directly comparable, even if all other variables are the same. For experimenters this restriction is not major. They need only ensure that all experiments are conducted using the same array platform and protocol. However, platform effects pose a significant problem for the re-analysis of data from multiple microarray studies.

Researchers who perform high throughput gene expression assays often deposit their data in public databases such as ArrayExpress [11] and Gene Expression Omnibus (GEO) [12], the latter of which currently houses 630, 845 assays distributed among 9, 348 platforms. Heterogeneity of measurement platforms leads to challenges for the re-use of these large data sets, creating limitations for researchers wishing to combine them. Extensive effort has been directed toward assessing the reproducibility of differential expression measurements across different platforms. Several studies have found good agreement among gene expression profiles produced by different platforms [13-18], while other studies have had conflicting results [19-21]. Technical issues pertaining to such evaluations include homogeneity of RNA samples, consistency of experimental protocols, mapping of probes across platforms, and the statistical methods used to assess reproducibility (e.g. direct comparison vs. log ratios). Those studies in which good intra-platform reproducibility was achieved and log ratios were compared across platforms generally showed good inter-platform reproducibility for oligonucleotide-based arrays. One study focusing on probe mapping in particular found that reproducibility between Affymetrix and cDNA platforms could be substantially improved by sequence-based re-annotation [22], and another found that reproducibility was further improved by mapping probe sequences at the exon level [14]. More recent studies generally show better cross-platform reproducibility than earlier ones [23]. It seems clear that, at least under ideal conditions, differential expression analysis gives consistent results across platforms. It is therefore worth asking how data from different platforms might be combined in an analysis. Models and techniques exist for the meta-analysis of microarray data from multiple studies and platforms [24-27], and these have been applied extensively to investigate questions of biological interest [28-33].

Cross-platform normalization differs from meta-analysis; the former involves direct comparison between expression measurements obtained from different platforms while the latter combines the results of intra-platform comparisons at a higher level. While meta-analysis techniques are extremely useful tools, they are limited to combining the results of studies that have tested the same hypothesis or compared the same treatments, and can not easily be applied to the investigation of new hypotheses from existing data.

Cross-platform normalization methods have been developed for the combination of data sets collected using different microarray platforms. These methods are the Cross-Platform Normalization (XPN) method of Shabalin et al.[34], Distance Weighted Discrimination (DWD) [35], an Empirical Bayes (EB) method also known as ComBat [36], Median Rank Scores (MRS)[6], Quantile Discretization (QD) [6], Normalized Discretization (NorDi) [37], the Distribution Transformation (DisTran) [5], and a method known as Gene Quantiles (GQ), which was developed as part of the WebArrayDB service [38]. In addition to these specialized methods, Quantile Normalization (QN) [39], a method commonly employed for intra-platform normalization, has also been applied to cross-platform normalization [40]. Many of the specialized methods include or are based closely on QN. Online analysis services currently offering some of these methods include WebArrayDB [38], ArrayMining [41], and DSGeo [40]. In addition, QN is available as part of Bioconductor [42], and code for some methods can be obtained from their respective authors.

Cross-platform normalization could be a valuable resource to researchers. While several studies have employed it for microarray analysis [4-6,43], it has not achieved the popularity of meta-analysis methods for the integration of results across studies and platforms. The online services listed above have received a total of 28 citations as of this writing according Google Scholar. It is difficult to judge the number of relevant citations for many methods, as some have alternative uses to cross-platform normalization or are introduced in publications describing other techniques. The publication describing XPN does not introduce any other methods or experiments, nor does XPN have any obvious applications other than cross-platform normalization. That article has been cited 34 times since its publication in 2008 according to Google Scholar, with only nine of those citing papers satisfying a full text search for the string "XPN." Those wishing to perform cross-platform normalization face three major obstacles. Firstly, a choice must be made about which method or methods to employ. While the authors of each method have demonstrated their methods on at least one example data set, to our knowledge no empirical comparison of cross-platform normalization methods similar to ours is available. In particular, no third party empirical comparison has been attempted. The authors of XPN do provide a comparison of their method against several others [34], but their analysis was conducted on a more limited data set and does not make use of resampling or any other procedure to evaluate the robustness of their results. This is not necessarily a shortcoming of their paper, but merely the result of a difference in objectives. The authors presented a method, and it is left for others to provide an unbiased evaluation. Secondly, software for the selected method must be obtained and incorporated into a data analysis work-flow. The disunity of interfaces and software packages for cross-platform normalization makes this task quite difficult for researchers lacking advanced computer skills, especially for methods that are only available as part of an online service. Some methods also rely on proprietary software, which presents an additional obstacle to integration. Thirdly, current cross-platform normalization techniques are only applicable to a limited subset of data sets. All the methods listed above require that every treatment group or sample type be represented on each platform. If this restriction is violated then it becomes impossible to distinguish platform effects from treatment effects of interest, and the latter may end up being removed by normalization.

In this paper we provide a comparison of available methods based on the MicroArray Quality Control (MAQC) project [17] data set and a human sperm data set [44] containing data from multiple platforms (see Methods for details). Envisioning potential applications to large scale databases or classification problems, we restrict our attention to cross-platform normalization performed without knowledge of treatment groups, and we examine the consequences of differently sized and missing treatment groups for the most successful methods. We also investigate the consistency of platform effects across different experiments. We have assembled an R [45] package capable of performing all of the methods investigated with a unified interface and reasonable defaults for user selectable parameters. Our package makes it possible for researchers to easily incorporate cross-platform normalization into existing work-flows, especially work-flows based on R or Bioconductor, and to experiment with multiple techniques without significant extra effort. Our package is available from the Comprehensive R Archive Network (CRAN, [46]). We explore a possible solution to the third difficulty above and show that it is insufficient in some cases.

## Results and Discussion

### Initial evaluation

**Figure 1:**
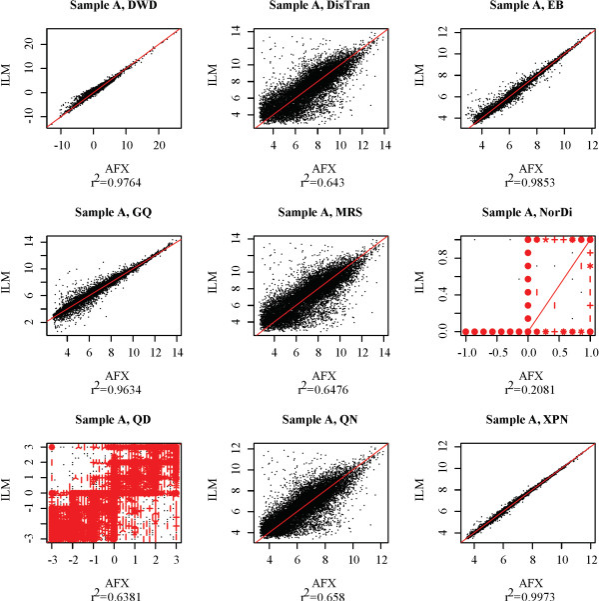
**Mean-mean plots for MAQC group A ILM and AFX data after cross-platform normalization**. Scatter or sunflower plots for MAQC treatment group A for each normalization method for the ILM and AFX data. Mean expression level of sample A assays on the AFX platform is plotted against mean expression level on the ILM platform. Plot titles indicate the cross-platform normalization method performed. Red lines are the line *y *= *x*, provided for visual comparison. Sub-titles indicate the *r*^2 ^value for the plot.

As an initial evaluation of the nine cross-platform normalization techniques, we applied each to a subset of the MAQC data set. Seven assays were selected at random from each of the four treatment groups, A, B, C, and D, included in the MAQC experiment from the Illumina (ILM) and Affymetrix (AFX) platform groups. The resulting reduced data set was then subjected to cross-platform normalization. To visually assess the effectiveness of each normalization technique, mean-mean expression scatter plots (or sunflower plots for methods with discrete output) were produced for each treatment group and each normalization method. Figure 1 shows mean-mean plots for treatment group A. Plots for treatment groups B, C, and D are included as additional files 1, 2, 3. Because each treatment group consists of technical replicates, all points on a mean-mean scatter plot should coincide with the line *y *= *x *under ideal circumstances. The closeness of the points to that line provides a measurement of inter-platform concordance. Throughout this work, we have used the squared Pearson correlation between the *x *and *y *values of these plots, denoted *r*^2^, as a statistical measure of inter-platform concordance.

**Figure 2:**
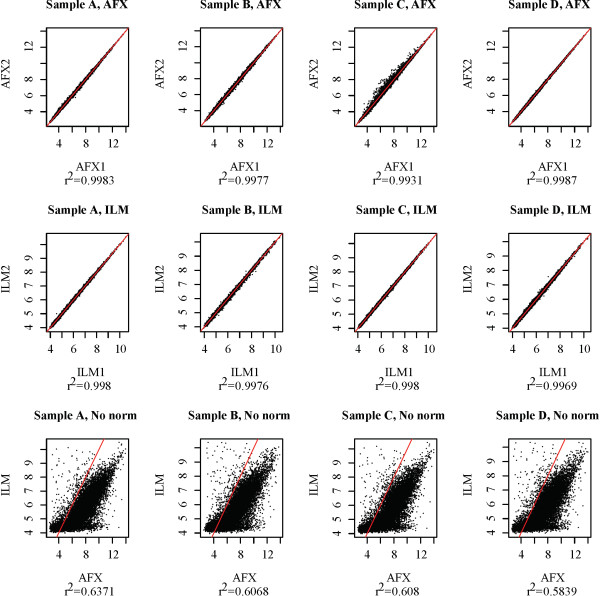
**Mean-mean plots for MAQC ILM and AFX data without cross-platform normalization**. Scatter plots for each MAQC treatment group. Single platform plots were produced from random non-overlapping subsets of seven assays each selected from the MAQC data set for that platform and treatment group. Red lines are the line *y *= *x*, provided for visual comparison. Sub-titles indicated the *r*^2 ^value for the plot.

Based on Figure 1, the cross-platform normalization methods are ranked by *r*^2 ^value as follows: XPN, EB, DWD, GQ, QN, MRS, DisTran, QD, and NorDi. There is a substantial gap between the fourth and fifth methods in the ranking, and another between the eighth and ninth. To better interpret these plots it is necessary to know the concordance that can be expected for technical replicates (replicates in which identical RNA samples were assayed) within a single platform and for technical replicates between two platforms. Ideally, the best cross-platform normalization methods will result in concordance levels similar to those obtained by technical replicates within a single platform. Figure 2 shows mean-mean plots for non-overlapping sets of assays selected from the data set for each platform, and a mean-mean plot for data from the two platforms without cross-platform normalization, for each of the four MAQC treatment groups. Intra-platform concordance is greater than .99 for all treatment groups and platforms, while inter-platform concordance is around 0.6 for all treatment groups. Figures 1 and 2 demonstrate that XPN, EB, DWD, and GQ substantially improve cross-platform concordance, while QN, MRS, DisTran, and NorDi provide little improvement and in some cases reduce concordance. The similar shapes displayed by the cross-platform scatter plots in Figure 2 suggest a consistency in platform effects among the four treatment groups. Such platform consistency is explored later on in this work.

**Figure 3:**
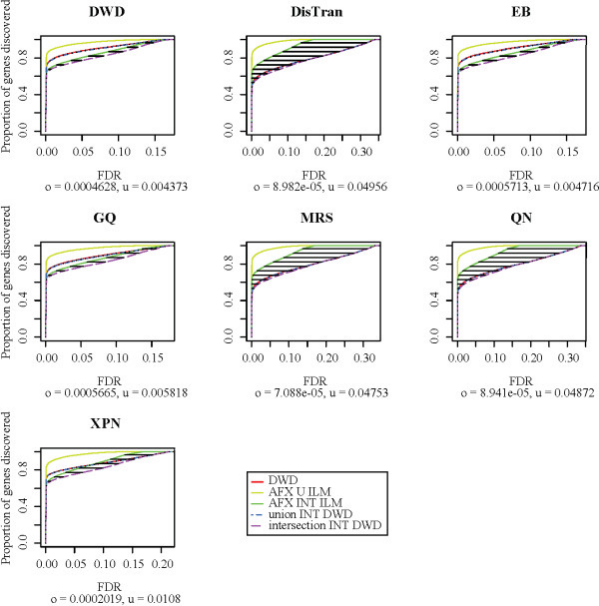
**ROC-like curves for the reduced MAQC data set**. ROC-like curves for the seven non-discrete cross platform normalization methods are applied to the reduced MAQC data set. Horizontal axes represent false discovery rate (FDR), while vertical axes represent the proportion of genes found to be differentially expressed between treatment groups A and B. Horizontal and vertical lines represent the areas used to compute under-detection (*u*) and over-detection (*o*), respectively, although no substantial areas of vertical lines are visible. The "union INT DWD" and "intersection INT DWD" curves represent the intersections (in the sense of gene sets) of the red curve with the yellow and green curves, respectively.

A trivial transformation could be devised to produce perfect concordance between platforms by the removal of all platform and treatment effects. However, such a transformation would be undesirable for cross-platform analysis because of the loss of treatment effects. To assess the degree to which treatment effects were retained during cross-platform normalization, we plotted Receiver Operating Characteristic (ROC)-like curves for seven of nine methods for treatment groups A and B from the reduced MAQC data set (Figure 3). NorDi and QD were excluded from further analysis because of their unsatisfactory scatter plots and the additional difficulty associated with discrete output. A true ROC curve plots true positives against false positives for a classifier [47]. The ROC-like curves used here show the proportion of genes classified as differentially expressed as a function of the estimated false discovery rate (FDR, see Methods for details). To provide a basis for comparison, we performed differential expression analysis using each platform separately to obtain a native differential expression set for each platform. An ROC-like curve was obtained from cross-platform data normalized by each method (the red curves in Figure 3), along with four additional curves for comparison. Two curves represent differentially expressed genes detected by either AFX or ILM (the union of native differential expression sets, yellow curves in Figure 3) and differentially expressed genes detected by both AFX and ILM (the intersection of native differential expression sets, green curves in Figure 3), and two more represent the intersections of genes detected by the cross-platform data set with the union (blue) or intersection (violet) of native differential expression sets from the individual platforms.

By comparing these curves we obtain statistics for over and under-detection of differentially expressed genes after cross-platform normalization as follows. Over-detection can be assessed by observing the difference between the ROC-like curve for genes detected with the cross-platform data set (the red curve) and the curve for the intersection of the cross-platform genes and the union of the single platform genes (the blue curve), which gives the genes detected using the cross-platform data set but not using either single platform data set. Under-detection can be assessed by observing the difference between the curve for the intersection of the two single platform gene sets (the green curve) and the curve for the intersection of all three gene sets (the violet curve), which gives the proportion of genes detected by both platforms individually but not by the cross-platform data set. In our further analyses, the areas between these two pairs of curves are used as statistics to measure over and under-detection, denoted *o *and *u*, respectively, of differentially expressed genes. These area statistics have the advantage of not depending on any arbitrary FDR cut-off, and to our knowledge have not been used previously.

It is not possible to rank cross-platform normalization methods by either one of the statistics *o *or *u *described above. For example, a method may bring *o *to nearly zero by removing all treatment effects. Such a method would not be desirable, and would result in a large *u*. Each of the statistics *o *and *u *guards against unrestricted optimization of the other. The *o *and *u *statistics are related to false positive and false negative rates, respectively, and in practice a good cross-platform normalization should strike a balance between the two.

Sorted from least to greatest under-detection, the methods are ordered: DWD, EB, GQ, XPN, MRS, QN, DisTran. Again, there is a major jump between the fourth and fifth ranked methods. When sorted instead by over-detection, the order is: MRS, QN, DisTran, XPN, DWD, GQ, EB, with a major discrepancy between the third and fourth ranked methods. Inspection of the ROC-like curves shows that the lower levels of over-detection among MRS, DisTran, and QN can be understood in terms of lower total detection of differentially expressed genes. By all three measurements, the seven methods cluster into two groups. The first group, made up of DWD, EB, GQ, and XPN, is characterized by higher concordance and over-detection and lower under-detection, while the second group, consisting of DisTran, MRS, and QN, is characterized by lower concordance and over-detection and higher under-detection. The ROC-like plots for the two groups are qualitatively different (Figure 3). In the first group, the curve for the cross-platform normalized data lies between the intersection and union curves for the native differential expression sets. In the second group, the curve for the normalized data always lies below the native intersection curve.

### Bootstrapping

#### Equally sized treatment groups

**Figure 4:**
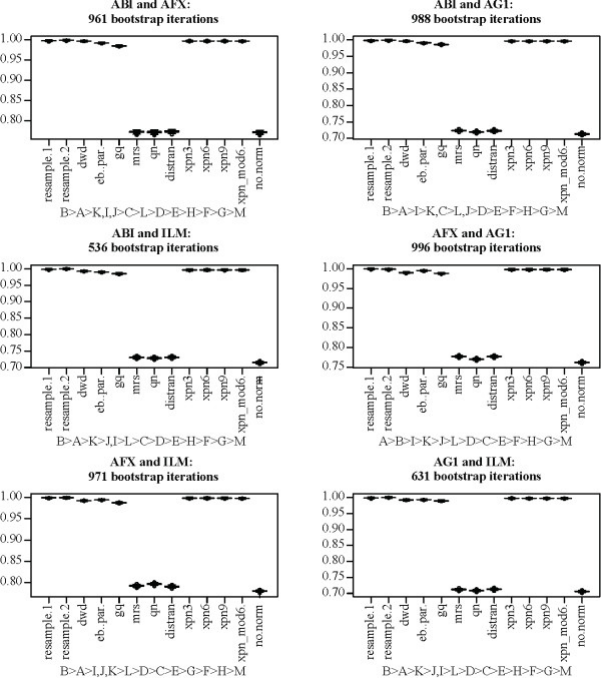
**Concordance (*r***^2^**) for normalization methods applied to the MAQC data**. Plot titles give the source platforms of the data being normalized. Boxes show the interquartile range and whiskers extend to an additional 1.5 times the interquartile range. Values outside the whiskers are plotted as circles. Notches are drawn such that non-overlapping notches are strong evidence of differing medians [61,45]. Subtitles show ranking of the methods. A: resample1, B: resample2, C: dwd, D: eb.par, E: gq, F: mrs, G: qn, H: distran, I: xpn3, J: xpn6, K: xpn9, L: xpn_mod6, M: no.norm. Inequalities in sub-titles are significant at the 0.5/*n*^2 ^level, where *n *is the number of methods in each sub-figure (including controls), by two-sided Mann-Whitney U-test. Commas indicate the difference in ranking is not significant. Numbers indicate the number of gene clusters used for XPN, e.g. xpn6 means XPN was performed using 6 gene clusters.

**Figure 5:**
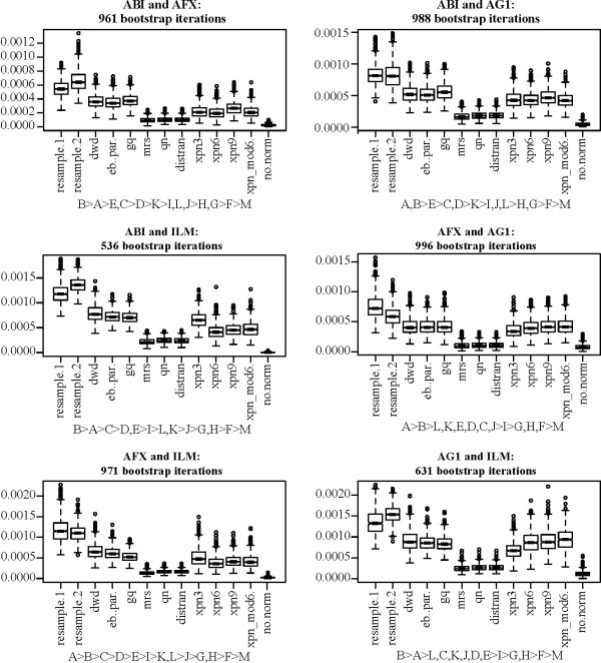
**Over-detection (***o***) for normalization methods applied to the MAQC data**. Plot titles give the source platforms of the data being normalized. Boxes show the interquartile range and whiskers extend to an additional 1.5 times the interquartile range. Values outside the whiskers are plotted as circles. Notches are drawn such that non-overlapping notches are strong evidence of differing medians [61,45]. Subtitles show ranking of the methods. A: resample1, B: resample2, C: dwd, D: eb.par, E: gq, F: mrs, G: qn, H: distran, I: xpn3, J: xpn6, K: xpn9, L: xpn_mod6, M: no.norm. Inequalities in sub-titles are significant at the .05/*n*^2 ^level, where *n *is the number of methods in each sub-figure (including controls), by two-sided Mann-Whitney U-test. Commas indicate the difference in ranking is not significant. Numbers indicate the number of gene clusters used for XPN, e.g. xpn6 means XPN was performed using 6 gene clusters.

**Figure 6:**
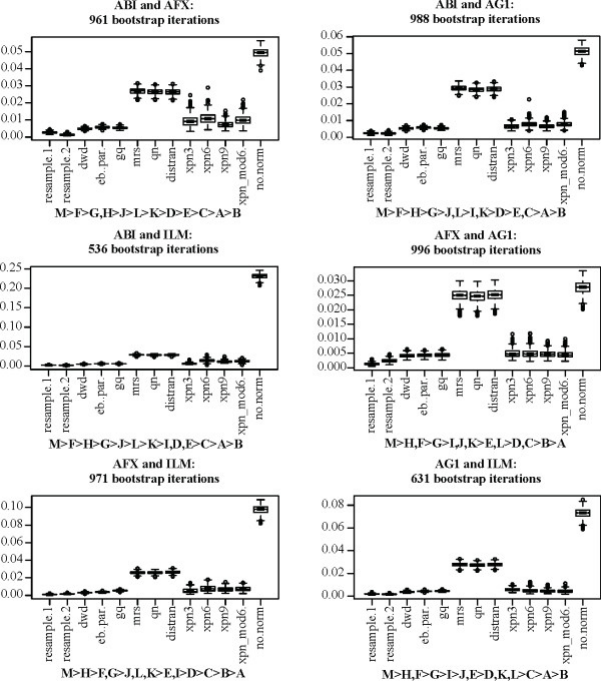
**Under-detection (*u*) for normalization methods applied to the MAQC data**. Plot titles give the source platforms of the data being normalized. Boxes show the interquartile range and whiskers extend to an additional 1.5 times the interquartile range. Values outside the whiskers are plotted as circles. Notches are drawn such that non-overlapping notches are strong evidence of differing medians [61,45]. Subtitles show ranking of the methods. A: resample1, B: resample2, C: dwd, D: eb.par, E: gq, F: mrs, G: qn, H: distran, I: xpn3, J: xpn6, K: xpn9, L: xpn_mod6, M: no.norm. Inequalities in sub-titles are significant at the .05/*n*^2 ^level, where *n *is the number of methods in each sub-figure (including controls), by two-sided Mann-Whitney U-test. Commas indicate the difference in ranking is not significant. Numbers indicate the number of gene clusters used for XPN, e.g. xpn6 means XPN was performed using 6 gene clusters.

Figures 1 and 3 provide a qualitative impression of the effectiveness of each method on the MAQC data set. For a quantitative assessment, we used the bootstrap to obtain distributions for the statistics *o*, *u*, and *r*^2^, defined above, for cross-platform normalization between the AFX and ILM MAQC data. Additionally, we included MAQC data from the Applied Biosystems (ABI) and Agilent one color (AG1) platforms, obtaining bootstrap distributions for all three statistics and all six combinations of the four platforms. For all bootstraps, a sample size of 15 assays was fixed for each treatment group for a total of 60 assays per bootstrap per platform. Figures 4, 5, and 6 show the distributions of *r*^2^, *o*, and *u*, for the seven non-discrete normalization methods. The XPN method includes a clustering step, and normalizations were performed with different clustering options. For comparison, distributions are also shown for data that have not been normalized (no.norm) and for resampled data from each individual platform (resample.1 and resample.2). The resampling trials simulate the result of performing an identical experiment again using each each platform separately. The resampled and non-normalized distributions serve as positive and negative controls, respectively, for successful cross-platform normalization.

In terms of concordance (Figure 4), results largely agree with our initial evaluation. Methods can be divided into the same two groups. The rankings of DWD, EB, GQ, and XPN fluctuate depending on the platforms, with one of the XPN methods always showing the highest ranking. XPN, DWD, EB, and GQ all perform near the level of the resampling controls, while MRS, QN, and DisTran perform near the level of the non-normalized cross-platform control. Over detection (Figure 5) showed the same pattern as in our initial comparison. We had stated that the reduced over-detection of MRS, QN, and DisTran may be due entirely to the reduced level of total detection for those methods. Here we show that the increased over-detection of XPN, DWD, EB, and GQ is still below the level of the resampling controls regardless of platform. Under-detection (Figure 6) was near but somewhat above the resampling controls for XPN, DWD, EB, and GQ for all platform combinations. MRS, QN, and DisTran fluctuated together depending on platform, always with a higher level than the other methods, and sometimes near the level of the negative control. DWD always showed the lowest level of under-detection, while an XPN variant was always the highest out of XPN, DWD, EB, and GQ. Variance of under-detection for the XPN methods appears markedly higher than for DWD, EB, and GQ. A Brown-Forsythe Levene-type test based on the absolute deviations from the median [48] showed the pooled variance of the XPN methods differed from that of DWD, EB, and GQ at a significance level of less than 1*e *- 20.

**Figure 7:**
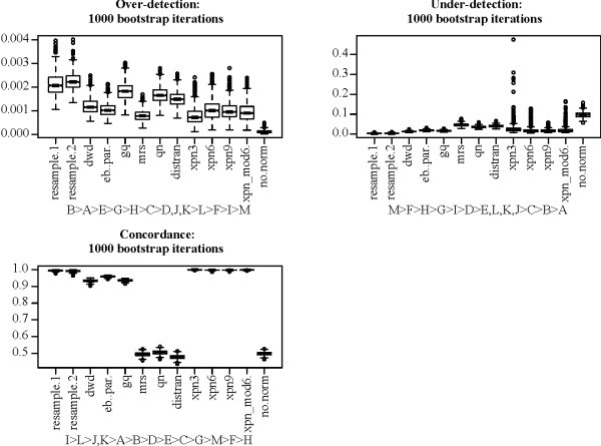
**Normalization methods applied to the human sperm AFX and ILM data**. Boxes show the interquartile range and whiskers extend to an additional 1.5 times the interquartile range. Values outside the whiskers are plotted as circles. Notches are drawn such that non-overlapping notches are strong evidence of differing medians [61,45]. Subtitles show ranking of the methods. Inequalities in sub-titles are significant at the .05/*n*^2 ^level, where *n *is the number of methods in each sub-figure (including controls), by two-sided Mann-Whitney U-test. Commas indicate the difference in ranking is not significant. Numbers indicate the number of gene clusters used for XPN, e.g. xpn6 means XPN was performed using 6 gene clusters.

The MAQC data set is unusual in that it contains a large number of technical replicates. The human sperm data set contains more biological than technical replicates and is more representative of data sets encountered in biological research. We performed the same bootstrapping analysis on the AFX and ILM human sperm data (Figure 7). Again, treatment group sizes were held fixed at 15 for both platforms for data obtained from both normal (N) and teratozoospermic (T) individuals. Results for concordance were similar to the MAQC data set, except that XPN outperformed the resample controls. All methods again showed over-detection levels below those of the resampling controls. DWD again showed the lowest level of under-detection, followed by GQ and EB. Previous trials of XPN showed levels similar to those of MRS, QN, and DisTran when more than 10 gene clusters were used (results not shown). XPN distributions for under-detection again showed higher variance than for DWD, EB, and GQ, and the difference was significant at the 1*e *- 20 level.

Over all, some results were consistent for all data sets tested. DWD, EB, GQ, and XPN outperform MRS, QN, and DisTran in both concordance and under-detection, with DWD showing the lowest over-detection and XPN the highest concordance in all cases. Over detection never exceeded levels observed in resampling controls, even for the non-normalized control, which in fact showed no over-detection. Because all treatment groups were the same size for both platforms, platform effects would be expected to cancel out when comparing treatment groups. High variance within each treatment group due to platform effects explains the under-detection seen in the negative control. The performance of MRS, QN, and DisTran varied with the data sets tested. MRS, QN, and DisTran generally performed poorly. However, they still outperformed the negative control in the sperm data set, which is consistent with past successful applications of those methods.

#### Unequally sized treatment groups

**Figure 8:**
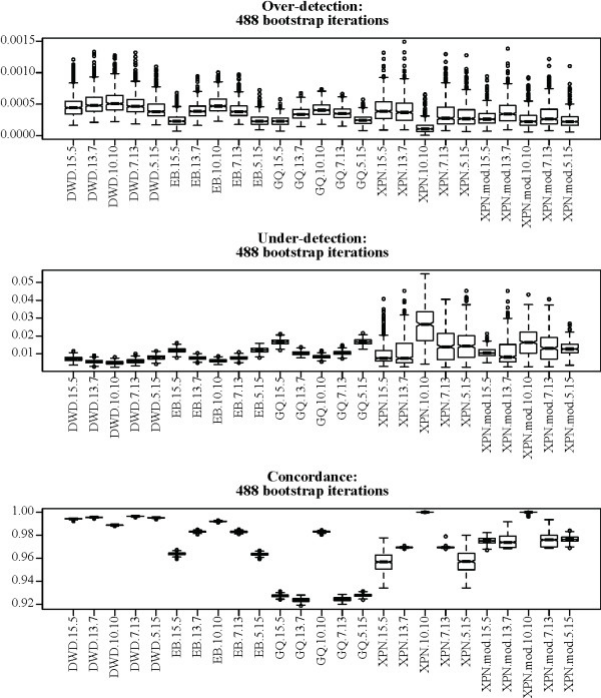
**Normalization methods applied to the MAQC data with unequal treatment groups**. Labels on the *x*-axis indicate method names and the treatment group sizes. Numbers indicate the sizes of treatment groups A and B, respectively, on the AFX platform and B and A, respectively, on the ILM platform, in terms of number of assays. For example, the label "method.*m.n*" indicates that the method "method" was applied to a data set containing *m *group A AFX assays, *n *group B AFX assays, *m *group B ILM assays, and *n *group A ILM assays. Boxes show the interquartile range and whiskers extend to an additional 1.5 times the interquartile range. Values outside the whiskers are plotted as circles. Notches are drawn such that non-overlapping notches are strong evidence of differing medians [61,45].

Four cross-platform normalization methods, DWD, EB, GQ, and XPN, showed satisfactory performance when evaluated using data sets with balanced treatment group sizes. Applications in which treatment group sizes are not consistent across platforms can easily be envisioned. We evaluated the four successful cross-platform normalization methods again using the MAQC data set with the same bootstrapping procedure. This time only data from treatment groups A and B were included, and the number of A and B samples was not necessarily equal between the two platforms. Five trials were performed for each method using different combinations of sample sizes: 15 A, 5 B for AFX and 5 A, 15 B for ILM; 13 A, 7 B for AFX and 7 A, 13 B for ILM; 10 A, 10 B for AFX and 10 A, 10 B for ILM; 7 A, 13 B for AFX and 13 A, 7 B for ILM; and 5 A, 15 B for AFX and 15 A, 5 B for ILM. Results indicate that DWD is the most robust to sample size differences, but that such differences have some effect on all methods (Figure 8). The under and over-detection statistics for XPN responded differently than for the other methods. Under-detection decreased for XPN as treatment group size disparity increased, and over-detection increased. For all other methods, the opposite was observed. All methods but DWD showed substantially decreased concordance with increasing treatment group size disparity. Our modified clustering procedure reduced this effect in XPN to some extent.

#### Missing treatment groups

**Figure 9:**
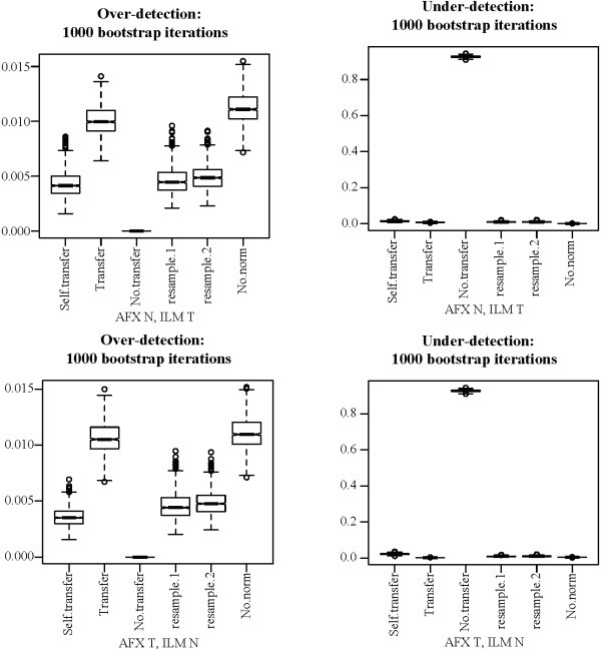
**DWD applied to human sperm data with missing treatment groups**. One treatment group was removed from each platform's data set before DWD was performed. Subtitles indicate the groups retained. Scrambled: Same as transfer, but parameters were randomly re-ordered before being used for cross-platform normalization. Self.transfer: Full human sperm data set was used for training. Transfer: MAQC data set was used for training. No.transfer: No additional training set was used. Boxes show the interquartile range and whiskers extend to an additional 1.5 times the interquartile range. Values outside the whiskers are plotted as circles. Notches are drawn such that non-overlapping notches are strong evidence of differing medians [61,45]. Differences between all pairs are significant at the .05/*n*^2 ^level, where *n *is the number of methods in each sub-figure (including controls), by two-sided Mann-Whitney U-test.

All cross-platform normalization methods studied share a common strategy. First, a set of parameters is determined from the data. Those parameters are then used to transform the data to remove platform effects. The parameters fitted from the data provide an estimate, in one form or another, of the platform effects present in the data set. Cross-platform normalization is only possible when all treatment groups are represented on both platforms. Otherwise, it becomes impossible to distinguish platform effects from treatment effects. Relaxation of this restriction would improve the usefulness of cross-platform normalization to researchers wishing to apply it. It may be possible to overcome this limitation by determining platform effects ahead of time using a separate data set, and then applying those parameters to the data set one wishes to transform. This possibility depends on the consistency of platform effects across different studies and treatment groups. To evaluate whether such consistency exists, we attempted to use parameters derived from the MAQC data set to perform DWD on the human sperm data set, first removing all N assays from the AFX data and all T assays from the ILM data, and then the reverse. DWD was selected for this experiment because of its success in previous trials and because of the simplicity of its model While concordance statistics could not be produced for this data set because data from each treatment group were represented on only one platform, we were able to evaluate both over-detection and under-detection using the same bootstrapping procedure as before (Figure 9). Sample sizes were again fixed at 15 for both platforms and treatment groups. "Self.transfer" indicates that the human sperm data set, rather than the MAQC, was used as a training set, which resulted in successful cross-platform normalization. "No.transfer" indicates that the missing treatments data set was used alone with no additional training data, which resulted in the removal of all treatment effects. The "Transfer" data set, for which the MAQC data set was used as a training set, showed over-detection near the level of non-normalized data, indicating that the MAQC platform effects, as estimated by DWD, differ substantially from those of the human sperm data set. The difference here does not imply that there is no consistency in the properties of different platforms, but merely that if such consistency exists then DWD is not sufficient to take advantage of it.

#### Exploring platform effects

**Figure 10:**
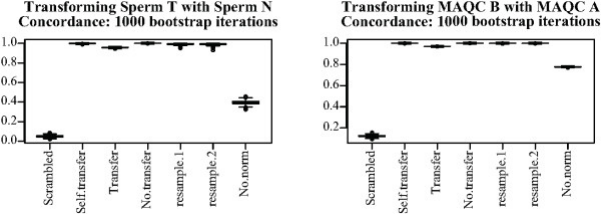
**DWD platform parameter transfer**. One treatment group was used as a training set for DWD to transform another treatment group in the MAQC and human sperm data sets. Titles indicate treatment groups used. Scrambled: Same as transfer, but parameters were randomly re-ordered before being used for cross-platform normalization. Self.transfer: Full human sperm data set was used for training. Transfer: Indicated treatment group was used as a training set. No.transfer: No additional training set was used. Boxes show the interquartile range and whiskers extend to an additional 1.5 times the interquartile range. Values outside the whiskers are plotted as circles. Notches are drawn such that non-overlapping notches are strong evidence of differing medians [61,45].

The inconsistency between platform effects in the MAQC and human sperm data sets may be due to differences in data processing or experimental protocols. Because of the age of the human sperm data set, its authors were unable to give any details regarding their exact procedures. To further explore the consistency of platform effects for different treatments, we tried using the MAQC A data to transform the MAQC B data and using the human sperm N data to transform the T data. Again we used the same bootstrapping procedure with 15 assays in each treatment group. Results (Figure 10) show reduced but high concordance compared to non-normalized data and to "scrambled" normalization, in which the location parameters used by DWD were randomly re-ordered after being estimated from the training set. Because the data being transformed contained only one treatment group, over and under-detection could not be assessed.

**Figure 11:**
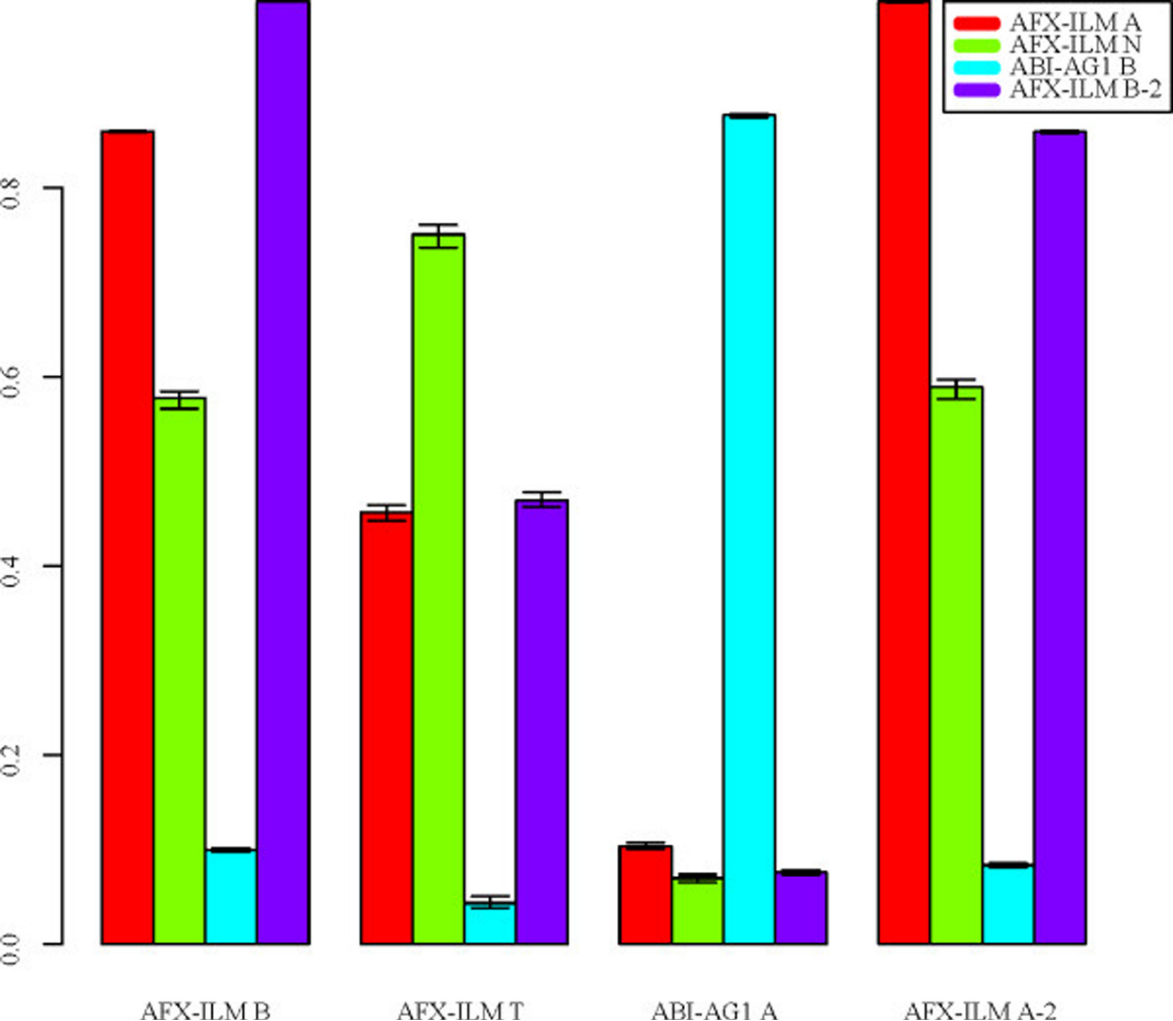
**Correlations between DWD parameters**. DWD parameters were obtained for each treatment group. Bars represent median correlation values between DWD parameters and whiskers represent interquartile ranges. AFX-ILM A-2 and B-2 represent independent resamples of the A and B data. Results represent 100 bootstrap iterations.

Figures 9 and 10 indicate that there is some consistency in platform effects between treatment groups within the same study, but possibly less between the MAQC and human sperm studies. We directly assessed the correlation between platform effects for different treatments, studies, and platforms again using DWD. Figure 11 shows correlations obtained between DWD parameters for 16 pairs of data sets. Each set shows the highest correlation with resampled data for the same treatment, study, and platform pair if present, the next greatest with data from a different treatment and the same study and platform pair, the next greatest with data from the same platform pair but different treatment and study, and the least with data from another platform pair regardless of treatment and study.

### Theoretical perspective

The four best performing methods, DWD, EB, GQ, and XPN, model platform effects using location parameters, while the other five methods do not include location shifts. The representation of platform effect as a location parameter is somewhat consistent with a physical interpretation of the microarray hybridization process. The hybridization process can be coarsely described by the chemical equation

(1) $$p+g\begin{array}{c} k_{+}\\ ⇌\\ k_{-} \end{array}h,$$

where *p*, *g*, and *h *are the probe, the gene or transcript target, and the hybridized transcript, respectively, and *k*_+ _and *k*_- _are rate constants. For relatively large target concentrations, the signal for a hybridized probe at equilibrium is

(2) $$S=\frac{Ig_{0}p_{0}\frac{k_{+}}{k_{-}}}{1+\frac{k_{+}}{k_{-}}g_{0}},$$

where *S *= *Ih *is measured signal, *g*_0 _is the initial concentration of target RNA or DNA, *p*_0 _is related to the initial number of available probe sites, and *I *is related to the intensity of the marking mechanism associated with each target DNA or RNA molecule. The parameter *I *should be thought of as the intensity of the label on each target molecule. The number of target molecules present at the probe site multiplied by the intensity of each gives the total intensity at that site. Model (2) has previously been applied to Affymetrix GeneChip^© ^data [49,50]. Under this model, the log of the measured signal is given by

(3) $$\log\left(S\right)=\log\left(g_{0}\right)+\log\left(Ip_{0}\frac{k_{+}}{k_{-}}\right)-\log\left(1+g_{0}\frac{k_{+}}{k_{-}}\right).$$

**Figure 12:**
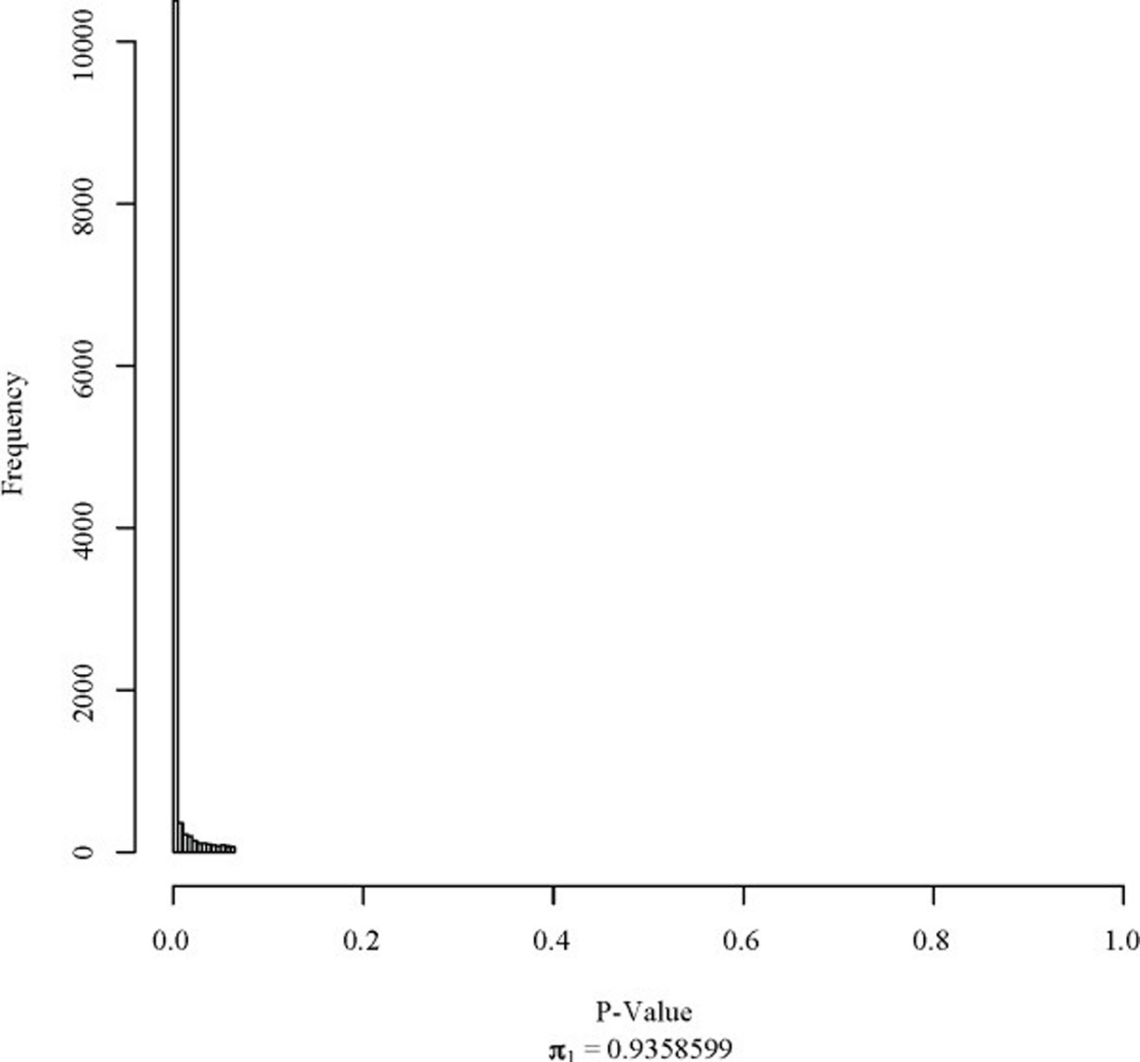
**P-values for ANOVA**. Histogram of *p*-values for treatment-platform interaction terms of model (4).

The parameters *k*_+_, *k*_-_, *I*, and *p*_0 _are determined by the microarray platform and experimental conditions such as hybridization temperature. Only *g*_0 _is related to gene expression. Assuming experimental conditions are about the same for all users of a given platform, a statistical model of log signal intensity for a particular probe might be

(4) $$y_{ijk}=T_{i}+P_{j}+C_{ij}+\varepsilon_{ijk},$$

where *y_ijk _*is the log signal value for gene *i*, treatment *j*, and repetition *k*; *T_i_*, *P_j_*, and *C_ij _*are treatment, platform, and treatment-platform interaction effects, respectively; and *ε_ijk _*is a random variable associated with repetition. Equation (4) is more general than, but fully consistent with, equation (3). The presence of a treatment-platform interaction term can be tested by analysis of variance (ANOVA). We performed ANOVA using model (4) for each gene separately on ABI, AFX, AG1, and ILM data with treatment groups A, B, C, and D from the MAQC data set, using the lm function of the R stats package [45]. Figure 12shows the distribution of *p*-values for the null hypothesis (*H*_0_) that *C_ij _*= *C *for all treatments and platforms. Using the qvalue package [51], we were able to estimate the proportion of genes, *π*_0_, for which *H*_0 _is true, and subsequently the proportion of genes, *π*_1_, for which *H*_0 _is false from the distribution of *p*-values using the method of Story and Tibshirani [52]. Results indicate that treatment-platform interaction effects exist for 93.59% of genes, or about 11, 315 genes out of 12, 091 total. Because not all genes are differentially expressed, it is to be expected that some genes show no interaction effect. If there is only one treatment group, platform and interaction effects don't need to be distinguished. Both *P *and *C *terms can be removed by a single location shift. For larger numbers of samples, the interaction term cannot be fully removed by a single location shift. An optimal location shift, in the least squares sense, for the *m *treatment case is

(5) $$y_{ijk}^{*}=y_{ijk}-\eta_{j}$$

(6) $$\eta_{j}=P_{j}+\frac{\sum_{i=1}^{m}n_{ij}C_{ij}}{\sum_{i=1}^{m}n_{ij}}$$

where $y_{ijk}^{*}$ is the corrected value and *η_j _*is the location shift used to correct for platform effects. The values *n_ij _*are the number of repetitions for treatment *i *and platform *j*, and *m *is the total number of different treatment groups. It is assumed that the true values of the effects are known. In practice, *η_j _*will be an estimate based on the data.

None of the methods studied here make explicit use of a least squares optimal location shift to remove platform effects. Rather, this optimal location shift is used to illustrate the limitations of modeling platform effects as location parameters under model (1). The prospect of determining a location shift for a particular pair of platforms using one data set and applying that shift to another data set is complicated by the presence of interaction effects in equation (6). By equations (5) and (6), the difference between two treatment group averages in a two platform data set after the application of a location shift derived from another two-platform data set, assuming equally sized treatment groups, is

(7) $${ȳ}_{1\cdot \cdot }^{*}-{ȳ}_{2\cdot \cdot }^{*}=T_{1}-T_{2}+\frac{1}{2}\overset{2}{\underset{i=1}{\sum }}\overset{2}{\underset{j=1}{\sum }}{\left(-1\right)}^{j-1}\left(C_{ij}-C_{ij}^{♢}\right)$$

where $C_{ij}^{♢}$ are the interaction terms from the training data set. Depending on the values of *C_ij _*and $C_{ij}^{♢}$, transference of parameters could result in the increased over-detection seen in our missing treatment group experiment. Other causes are also possible, including differences in scanners or image processing procedures.

Uniformity of platform effects across different experiments is consistent with the model (1). Additivity is not, although empirically it appears to be a useful approximation, as indicated by the relative success of the location based methods. The accuracy of that approximation may be reduced, however, when employing a separate training set to estimate platform effects. This reduction was observed in our analyses, in particular in Figures 9 and 10. Model (1) is a sufficient explanation for all of our observations, which suggests it or the more general equation (4) may provide a good basis for an improved cross-platform normalization method that addresses the issue of non-identical treatment groups across platforms. However, fitting such a model would require a data set containing multiple matched treatment groups for any pair of platforms on which it could be used.

### Software

To facilitate the application of cross-platform normalization by other researchers, we packaged all the implementations used in this work, including those obtained from other authors, using the R package mechanism. Our package, CONOR, includes documentation and provides a common interface for all methods, along with reasonable defaults for user-selectable parameters. The package can be downloaded at http://alborz.sdsu.edu/conor and is available from CRAN.

## Conclusions

Of the four methods capable of successful cross-platform normalization, DWD showed the least loss of treatment information and XPN showed the greatest inter-platform concordance, although the latter was sometimes in excess of resample controls and might be interpreted as a slight over-correction. DWD was the most robust to variations in treatment group sizes between the two platforms. This result is somewhat surprising because XPN incorporates an assay clustering step designed to correct for such variations [34]. Our clustering variant showed reduced sensitivity to treatment size disparity, and it is possible that further improvement to the clustering step of the XPN algorithm could result in improved robustness. It is no surprise that GQ and EB, which do not account for treatment group disparities, suffered reduced performance under such conditions. In general, those methods that employ location shifts (DWD, EB, GQ, and XPN) outperformed those that do not, and the performance of the methods that do not include such shifts was quite unsatisfactory. Many of those methods were not originally designed for cross-platform normalization, and their failure to accomplish such normalization does not imply that they are insufficient to their other uses. The EB and XPN methods make use of distributional assumptions about the data. XPN uses a normally distributed residual for maximum likelihood estimation, while EB employs a complicated model including parametric prior distributions. For the log-transformed microarray data used in this study, these methods performed well. However, the distributional assumptions of these methods must be valid for their performance to be assured. When performed on non-log transformed data, for example, these methods fail to produce good results. In cases in which normality is in doubt, appropriate transformations (such as log transformation) should be employed. Our analyses considered cross-platform normalization in the absence of treatment group information. It is possible that superior methods to those investigated could be devised that make use of treatment information. The EB method is already able to accommodate treatment group membership information if it is available.

Our experiment in normalizing the human sperm data set using parameters derived from the MAQC data set shows that there may be some consistency in platform effects across different treatments. Larger differences between platform effects in the MAQC and human sperm studies may have resulted from differences in protocols between the two studies. However, they may also have resulted from larger disparity between gene expression patterns in sperm and those of other human tissues, in which case a more complicated model of platform effects might resolve the difference. Ideally, data sets like the MAQC project's could be used as "Rosetta Stones" for gene expression platforms, allowing data collected on one platform to be translated to be comparable with data from another platform regardless of treatment group disparities. This work has shown that a model including treatment-platform interaction terms will be required for such a system to be effective, and further investigation is required before such a system can be realized.

Next generation sequencing technology is replacing microarrays for the measurement of gene expression. The types of platform effects present in microarray data are probably not relevant for sequencing-based expression data. Nevertheless, existing databases, as well as microarray experiments that may be performed in the near future, represent a substantial resource. Cross-platform normalization has the potential to become a valuable tool for gene expression research by allowing researchers to combine and analyze existing data together with new data or in new contexts. While at least nine methods are currently available, we've shown that only four of those methods provide reasonable results on the MAQC and human sperm data sets. Although researchers are encouraged to draw their own conclusions based on their particular needs, two methods emerged as most effective. *In cases in which false positives are to be preferred over false negatives, or in which treatment group sizes are not equal for the various single platform data sets, DWD is the recommended cross-platform normalization method because of its lower under-detection and improved robustness. In cases in which false negatives are to be preferred to false positives, and for use with classifiers, XPN is recommended because of its superior cross-platform concordance. If there is doubt as to which situation applies, DWD is recommended, but there is no reason that multiple techniques could not be employed and the results compared*. The existence of the CONOR package will make the latter option particularly painless. In cases in which treatment groups are missing from one platform or another, the DWD-based procedure described here is the only currently available method, but care must be taken to ensure that training data have sufficiently similar transcription profiles to the data being transformed.

Cross-platform normalization of large or complex datasets in which treatment groups are missing will require improved models of treatment-platform interaction effects. Future work should include the modification of XPN to improve robustness and allow for the use of a separate training set, as well as the investigation of other models of platform effects. This study has not examined every platform available. In particular, no cDNA arrays were included, and the effectiveness of cross-platform normalization with such arrays should be investigated before those methods are applied. While the types of platform biases present in microarrays are not relevant to sequencing based assays, it is worth investigating the effects that different extraction, amplification, and sequencing methodologies have of such measurements, as well as how data from sequencing-based assays might be combined with microarray data. While the availability of an R package does much to make these methods accessible, there are some researchers who are not comfortable with the R command-style interface. For those researchers, a GUI application or web interface integrating all of those methods may be beneficial, although web interfaces are already available for some methods. If the transfer of platform parameters between data sets is improved, a web-based database of such platform parameters that could be integrated into cross-platform analyses would be extremely useful.

## Methods

### Data preparation

The MAQC data set [17] contains assays of two distinct RNA samples, Stratagene Universal Human Reference RNA (treatment group A) and Ambion Human Brain Reference RNA (treatment group B), along with 3:1 (group C) and 1:3 (group D) mixtures of the two. Each sample was assayed repeatedly at two or more independent sites for each platform included. The platforms used in our analyses were the Applied Biosystems Human Genome Survey Microarray V2.0, the Affymetrix HG-U133 Plus 2.0 GeneChip^©^, the Agilent Whole Genome Oligo Microarray G4112A, and the Illumina Human-6 BeadChip 48k v1.0. These platforms were selected based on the availability of data and the variety of probe lengths, manufacturing techniques, and detection methods employed (see Table 1).

The human sperm data set [44] contains assays of mRNA from sperm obtained from normally fertile men (group N) and teratozoospermic men (group T), assayed using the same AFX and ILM arrays as were used in MAQC, as well as another Illumina array, which was not used in this study. While some individual samples were assayed on both the AFX and ILM platforms, assays in the N or T group of each platform represent biological rather than technical replicates. Each assay comes from a unique sample and individual.

The MAQC and human sperm data were obtained from GEO [GEO: GSE5350 and GSE6969]. The data set for each microarray platform was subject to different preprocessing. Preprocessing did not make use of treatment group membership, as such knowledge would not be available or easily incorporated for some applications. For all data sets except ABI, quantile normalization was used as an intra-platform normalization strategy to remove assay effects. Quantile normalization is a well established and simple method for intra-platform normalization. While other methods are available [39], we found quantile normalization to be sufficient for our purposes. All expression data were subject to log transformation, and it is worth noting that most cross-platform normalization methods failed to perform well on data that was not log transformed in our initial trials (results not included). Those methods that failed to perform on non-log data were those that employed statistical models, and their poor performance is likely the result of violations of the distributional assumptions of those models.

#### Applied Biosystems

The ABI data were downloaded already normalized from GEO using the GEOquery package [53] from Bioconductor. These data were normalized by the MAQC authors using the Expression Array System Software suite from Applied Biosystems, which implements a platform specific normalization sequence based on the specific properties of the ABI array and the 1700 Chemiluminescent Microarray Analyzer. The steps in this normalization sequence take advantage of co-localized control probes and signal to noise ratios obtained during image processing. Details can be found in the supplemental materials of the MAQC publication [17] and in the document entitled "User Bulletin: Applied Biosystems 1700 Chemiluminescent Microarray Analyzer" issued by ABI [54]. The downloaded data were natural log transformed before use in my analyses. It should be noted that the ABI data set was the only set not subjected to quantile normalization.

#### Affymetrix

Raw Affymetrix CEL files were pre-processed using the function justRMA from the affy package [55] of Bioconductor. For the experiments involving both MAQC and human sperm data, both sets were normalized together. For all other experiments, the MAQC and sperm data were normalized separately.

#### Agilent

The raw Agilent data were processed using the limma package [56] from Bioconductor. Background correction was performed using the "normexp" and "mle" options of the backgroundCorrect function. Quantile normalization was performed by the normalizeBetweenArrays function. Data from duplicate probes was then averaged and natural log transformation performed.

#### Illumina

Raw Illumina data were acquired from GEO in text format. The mean signal for each probe type was extracted and subjected to quantile normalization (provided by the normalize. quantiles function from Bioconductor's preprocessCore package) and natural log transformation. For the experiments involving both MAQC and human sperm data, both sets were quantile normalized together. For all other experiments, the MAQC and sperm data were subjected to separate quantile normalization.

#### Probe mapping

The MAQC publication [57] provides mappings for all platforms involved in the study for 12, 091 common genes as Supplementary Table five, and we used that mapping for all cross-platform normalization experiments for both the MAQC and human sperm data sets. The mapping was accomplished by BLASTing probe sequences against the human RefSeq database. A detailed BLAST protocol is also provided in the supplementary materials of the MAQC publication, which are available from the Nature Biotechnology website. The RefSeq database has been improved and updated since 2006, and some justification is required for the use of gene annotations that are presently more than four years old. While it is generally advisable to use the most recent annotations available when analyzing microarray data, little is to be gained in this case by repeating the mapping with a more recent version of RefSeq. The number of additional probes that could be mapped using the current version of RefSeq is likely to be very small, especially when considering that the arrays used were designed before the original mapping was produced and that the human genome was already well mapped by 2006. As the purpose of this study is to compare the various methods for cross-platform normalization, and not to discover genes of biological interest, we believe the MAQC mapping to be sufficient.

### Plots and statistics

#### Mean-mean plots and inter-platform concordance

Mean-mean expression scatter plots were produced by plotting the average expression value for each gene and treatment group on one platform against the average expression for the corresponding gene and treatment group of another platform. Treatment groups for MAQC data are equivalent to sets of technical replicates (since the same RNA pool was used for all assays), whereas treatment groups in the human sperm data set (samples obtained from normal and teratozoospermic individuals) represent biological replicates, although some technical replicates are included. Squared Pearson correlation between the *x *and *y *coordinates of each point in the mean-mean scatter plot was used as a statistic to measure inter-platform concordance. It has been pointed out by the editors that the concordance correlation coefficient is a more appropriate statistic for these purposes [58]. Unfortunately this error was brought to our attention after it was possible to make the change. We believe, however, that the conclusions of this work are not impacted by the use of the squared Pearson correlation for two reasons: firstly, that the mean-mean plots used in this study generally show a good fit to the line *y *= *x*, and secondly, that the conclusions drawn are supported by the other statistics used.

#### Differential expression and ROC-like curves

Differential expression was assessed using the p-value of a two-sided Welch's t-test as a statistic. A p-value was obtained for each gene, and the resulting list of p-values was transformed into a list of q-values using the q-value package [51], available from Bioconductor. The *q*-value for a particular feature (or gene) is defined as the proportion of false positives expected when calling all features on a list up to and including that one significant, where here the list in question is the list of *p*-values obtained from the t-tests [52]. The empirical cumulative distribution function (cdf) of the resulting list of q-values is then equivalent to the ROC-like curves presented. Similar curves have been used previously in the evaluation of methods for identifying differentially expressed genes [57].

The union and intersection curves were obtained by taking the empirical cdf of the gene-wise minimum and maximum, respectively, of the two q-value lists. Areas between curves were obtained by numerical integration using the trapezoid method, as implemented in the caTools package [59] available from CRAN. Curves were sampled at intervals of 0.001 on the FDR axis. Over detection was measured as the area between the ROC-like curve for differentially expressed genes detected using cross-platform normalized data and the intersection of that curve with the curve representing the union of genes detected using each platform independently. Under detection was measured as the area between the curve representing the intersection of genes detected using each platform independently and the intersection of that curve with the curve representing differentially expressed genes detected using cross-platform normalized data. The sample sizes were equalized for all curves during the resampling process.

### Bootstrapping

A smoothed bootstrapping procedure was used to obtain distributions for the concordance, over-detection, and under-detection statistics. The smoothing was accomplished through the addition of zero-centered Gaussian noise with a 0.1 standard deviation. Resampling was restricted by treatment groups. That is, for the MAQC data set, every bootstrap maintained the same proportion of data from samples A, B, C, and D. For the human sperm data set, the proportion of data from N and T samples was kept constant. Data used to produce the native ROC-like curves for each platform (and subsequent union and intersection curves) were produced by the same resampling and smoothing procedure, but with the sample size doubled for the relevant treatment groups in order to maintain the same total sample size for each ROC-like curve produced. Data for resampling (positive) control methods was generated in the same manner as the data for the native ROC-like curves, sampled independently to simulate repetition of the experiment on each platform separately. Computations were performed using R version 2.9.0 (2009-04-17) x86_64-redhat-linux-gnu on a Rocks 5.3 computing cluster. Bootstrapping results are available as additional file 4.

### Cross-platform normalization

R code was obtained for all methods if available. R functions for GQ, MRS, and QD were taken from the source code for WebArrayDB. Code for EB and NorDi was obtained from the original authors of those methods. QN was available from the preprocessCore package of Bioconductor. To the best of our knowledge, no R implementations of DWD, DisTran, or XPN existed prior to this work. Implementation of DisTran was based on a description of the method [5]. Because the DisTran method relies on treatment group information, which for the purposes of this comparison was not considered to be available, a *k*-means clustering step was added to estimate that information. The correct number of treatment groups was always used as the value of *k*. Implementation of DWD and XPN was based on the Matlab implementations of those methods provided by their authors. Both methods were tested on toy data sets to ensure agreement with the original implementations. For XPN, agreement was approximate due to the randomness inherent in the initial clustering step. DWD relies on the solution to a second order cone program (SOCP). No appropriate SOCP solver existed in R prior to this work, so a recent smoothing Newton method algorithm was implemented and employed [60]. Additional clustering features were added to XPN. In the original Matlab implementation, *k*-means clustering of assays was performed on data from both platforms together. If any cluster contained data from only one platform, clustering was simply repeated with a different set of initial centroids. To speed up the clustering process, an option to cluster data from the two platforms separately and match clusters based on the correlation of cluster centroids was added. XPN trials designated as "modified" or "mod" made use of this modified method.

Some methods require the user to select one or more parameters manually. XPN and DisTran both require the user to select how many assay clusters are to be used. In all cases, the actual number of treatment groups was used. XPN also requires the user to select the number of gene clusters and the number of iterations to perform. Values of 3, 6, and 9 were used for gene clusters as indicated in the results section. Where no value is indicated, three gene clusters were used. For all XPN trials, 30 iterations were performed as recommended by the original authors [34]. The EB method allows the user to select whether a parametric or non-parametric prior distribution is to be used. For all trials shown, the parametric prior was selected. Difficulties were encountered with the non-parametric option. NorDi requires the selection of *p*-value and alpha parameters, which were set at 0.01 and 0.05, respectively, in agreement with that method's use by its authors.

A detailed description of each method can be found in the references, with the exception of GQ. GQ is included as part of the WebArrayDB service [38] and was invented by the authors of that service, but has never been described in a publication. GQ is a two step process. First, the data are transformed by the MRS method. Second, the median expression value is calculated for each gene and platform combination in the MRS normalized data. The median expression values are then subtracted from the second platform data, and the first platform's medians are added to the second platform's data set, with the end result that each gene has the same median expression level on both platforms.

The EB method is capable of taking treatment group or other covariate information into account [36]. Because we are interested in applications in which such information may be unavailable, we did not utilize those capabilities in our analyses.

## Authors' contributions

JR located data, performed all programming and analyses, and wrote the manuscript. FV supervised all research, guided decision making with regard to methods, and edited the manuscript. Both authors contributed to the conception and design of the project. All authors read and approved the final manuscript.

## Supplementary Material

Additional file 1 **Mean-mean plots for MAQC treatment group B ILM and AFX data**. Click here for file

Additional file 2 **Mean-mean plots for MAQC treatment group C ILM and AFX data**. Click here for file

Additional file 3 **Mean-mean plots for MAQC treatment group D ILM and AFX data**. Click here for file

Additional file 4 **Raw bootstrap results for all statistics presented**. Click here for file
