# Supplementary material for: Empirical comparison of cross-platform normalization methods for gene expression data
Source: BMC Bioinformatics. 2011 Dec 7;12:467. doi: 10.1186/1471-2105-12-467 (PMC3314675; doi:10.1186/1471-2105-12-467)
Supplement: Additional file 1 — Mean-mean plots for MAQC treatment group B ILM and AFX data. [file 1471-2105-12-467-S1.pdf]

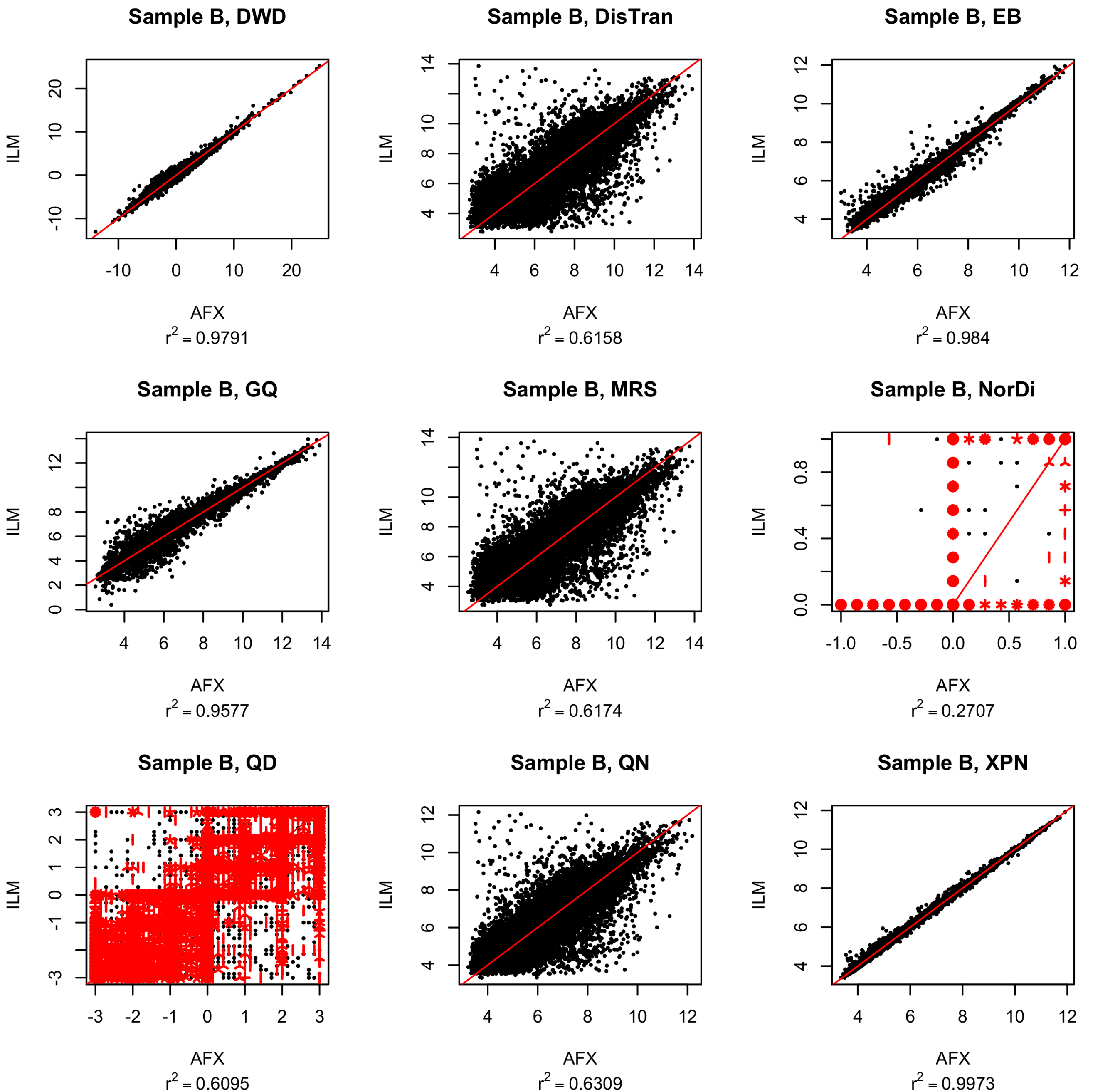
Sample B, DWD
ILM
AFX
-10 0 10 20
r2 = 0.9791
Sample B, DisTran
ILM
AFX
4 6 8 10 12 14
r2 = 0.6158
Sample B, EB
ILM
AFX
4 6 8 10 12
r2 = 0.984
Sample B, GQ
ILM
AFX
0 2 4 6 8 12
4 6 8 10 12 14
r2 = 0.9577
Sample B, MRS
ILM
AFX
4 6 8 10 12 14
r2 = 0.6174
Sample B, NorDi
ILM
AFX
0.0 0.4 0.8
-1.0 -0.5 0.0 0.5 1.0
r2 = 0.2707
Sample B, QD
ILM
AFX
-3 -1 0 1 2 3
-3 -2 -1 0 1 2 3
r2 = 0.6095
Sample B, QN
ILM
AFX
4 6 8 10 12
r2 = 0.6309
Sample B, XPN
ILM
AFX
4 6 8 10 12
r2 = 0.9973
