# Supplementary material for: Empirical comparison of cross-platform normalization methods for gene expression data
Source: BMC Bioinformatics. 2011 Dec 7;12:467. doi: 10.1186/1471-2105-12-467 (PMC3314675; doi:10.1186/1471-2105-12-467)
Supplement: Additional file 2 — Mean-mean plots for MAQC treatment group C ILM and AFX data. [file 1471-2105-12-467-S2.pdf]

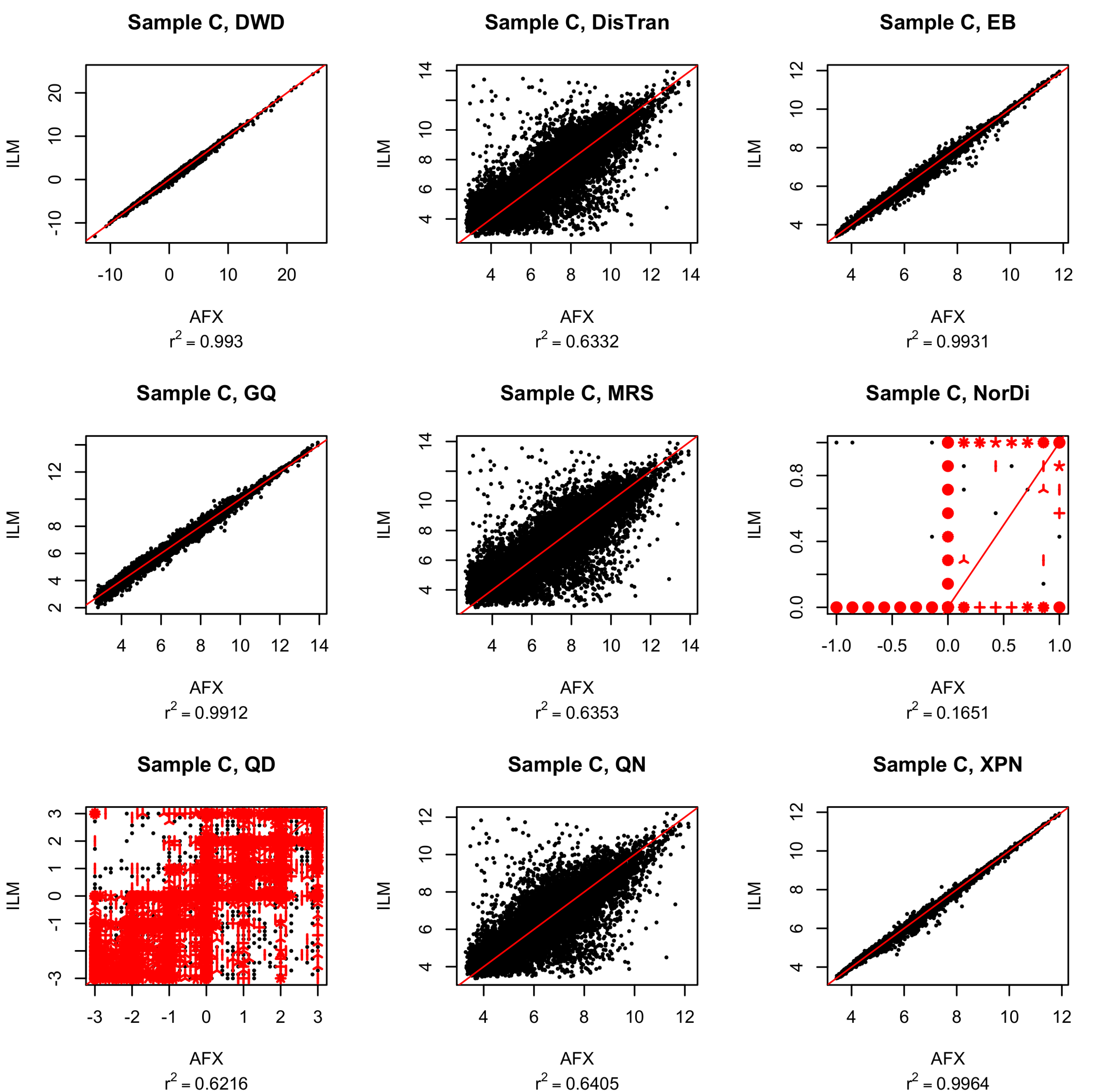
Sample C, DWD
ILM
AFX
$r^2 = 0.993$
Sample C, DisTran
ILM
AFX
$r^2 = 0.6332$
Sample C, EB
ILM
AFX
$r^2 = 0.9931$
Sample C, GQ
ILM
AFX
$r^2 = 0.9912$
Sample C, MRS
ILM
AFX
$r^2 = 0.6353$
Sample C, NorDi
ILM
AFX
$r^2 = 0.1651$
Sample C, QD
ILM
AFX
$r^2 = 0.6216$
Sample C, QN
ILM
AFX
$r^2 = 0.6405$
Sample C, XPN
ILM
AFX
$r^2 = 0.9964$
