# Supplementary material for: Empirical comparison of cross-platform normalization methods for gene expression data
Source: BMC Bioinformatics. 2011 Dec 7;12:467. doi: 10.1186/1471-2105-12-467 (PMC3314675; doi:10.1186/1471-2105-12-467)
Supplement: Additional file 3 — Mean-mean plots for MAQC treatment group D ILM and AFX data. [file 1471-2105-12-467-S3.pdf]

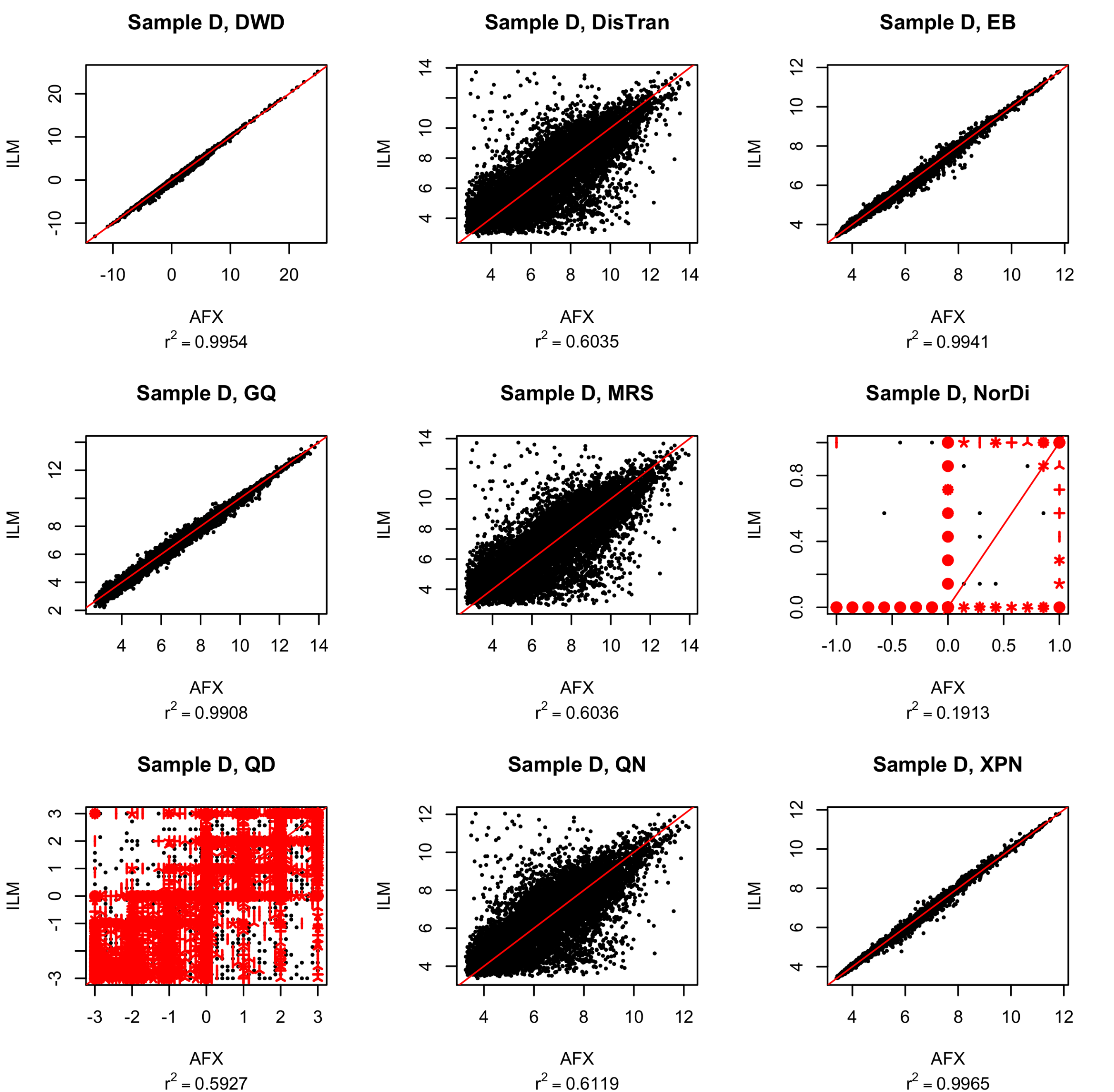
Sample D, DWD
ILM
AFX
r^2 = 0.9954
Sample D, DisTran
ILM
AFX
r^2 = 0.6035
Sample D, EB
ILM
AFX
r^2 = 0.9941
Sample D, GQ
ILM
AFX
r^2 = 0.9908
Sample D, MRS
ILM
AFX
r^2 = 0.6036
Sample D, NorDi
ILM
AFX
r^2 = 0.1913
Sample D, QD
ILM
AFX
r^2 = 0.5927
Sample D, QN
ILM
AFX
r^2 = 0.6119
Sample D, XPN
ILM
AFX
r^2 = 0.9965
